# Neuroprotective Properties of Coriander-Derived Compounds on Neuronal Cell Damage under Oxidative Stress-Induced SH-SY5Y Neuroblastoma and in Silico ADMET Analysis

**DOI:** 10.1007/s11064-024-04239-0

**Published:** 2024-09-19

**Authors:** Papitcha Jongwachirachai, Waralee Ruankham, Setthawut Apiraksattayakul, Saruta Intharakham, Veda Prachayasittikul, Wilasinee Suwanjang, Virapong Prachayasittikul, Supaluk Prachayasittikul, Kamonrat Phopin

**Affiliations:** 1 Center for Research Innovation and Biomedical Informatics, Faculty of Medical Technology, Mahidol University, Bangkok, 10700 Thailand; 2 Department of Clinical Microbiology and Applied Technology, Faculty of Medical Technology, Mahidol University, Bangkok, 10700 Thailand

**Keywords:** Neuroprotection, Antioxidant, Oxidative stress, Linalool, Linalyl acetate, Geranyl acetate

## Abstract

An imbalance between reactive oxygen species (ROS) production and antioxidant defense driven by oxidative stress and inflammation is a critical factor in the progression of neurodegenerative diseases such as Alzheimer’s and Parkinson’s. Coriander (*Coriandrum sativum* L.), a culinary plant in the *Apiaceae* family, displays various biological activities, including anticancer, antimicrobial, and antioxidant effects. Herein, neuroprotective properties of three major bioactive compounds derived from coriander (i.e., linalool, linalyl acetate, and geranyl acetate) were investigated on hydrogen peroxide-induced SH-SY5Y neuroblastoma cell death by examining cell viability, ROS production, mitochondrial membrane potential, and apoptotic profiles. Moreover, underlying mechanisms of the compounds were determined by measuring intracellular sirtuin 1 (SIRT1) enzyme activity incorporated with molecular docking. The results showed that linalool, linalyl acetate, and geranyl acetate elicited their neuroprotection against oxidative stress *via* protecting cell death, reducing ROS production, preventing cell apoptosis, and modulating SIRT1 longevity. Additionally, in silico pharmacokinetic predictions indicated that these three compounds are drug-like agents with a high probability of absorption and distribution, as well as minimal potential toxicities. These findings highlighted the potential neuroprotective linalool, linalyl acetate, and geranyl acetate for developing alternative natural compound-based neurodegenerative therapeutics and prevention.

**Supplementary Information:**

The online version contains supplementary material available at 10.1007/s11064-024-04239-0.

## Introduction

The risks of developing several chronic conditions (i.e., cardiovascular diseases, cancers, diabetes, and neurodegenerative diseases) are increasing worldwide due to a global aging society [1]. Neurodegenerative diseases (NDs) are a group of irreversible diseases with progressive loss of neurons leading to psychological, physiological, and behavioral changes of the patients. NDs are mostly asymptomatic at the early stages, while symptoms manifested in the late stages are considerably life-burden such as impaired cognitive function, impaired movements, or death in severe cases [2]. The two most common, Alzheimer’s (AD) and Parkinson’s (PD) diseases, have become one of the leading causes of death worldwide [3]. However, current clinically available drugs are only symptomatic treatment, but unable to cease or slow down the progression of the diseases [4]. Therefore, the development of the disease-modifying agents with protective potentials are essential for more effective management and prevention of NDs.

Reactive oxygen species (ROS) including superoxide, hydrogen peroxide (H_2_O_2_), and hydroxyl ions are important free radicals, in which their optimal levels and functions are required for several biological processes (i.e., protein proliferation, apoptosis, immunity, differentiation, and transcription) [5]. The increase in ROS levels, either by increasing the production or decreasing the antioxidant defense, can cause an imbalance between free radicals and antioxidants leading to oxidative stress (OS) and cellular damage [6]. The OS plays an important role in the pathogenesis of several diseases, including NDs. Over-accumulation of ROS can cause mitochondrial dysfunction, activation of pro-apoptotic pathways, and disruption of cellular homeostasis *via* oxidation of proteins, lipids, and nucleic acids, leading to neuronal cell loss which implicates the initiation and progression of NDs [7]. Accordingly, bioactive compounds possessing antioxidant activity have become prominent candidates for discovery of neuroprotective agents. 

**Fig. 1:**
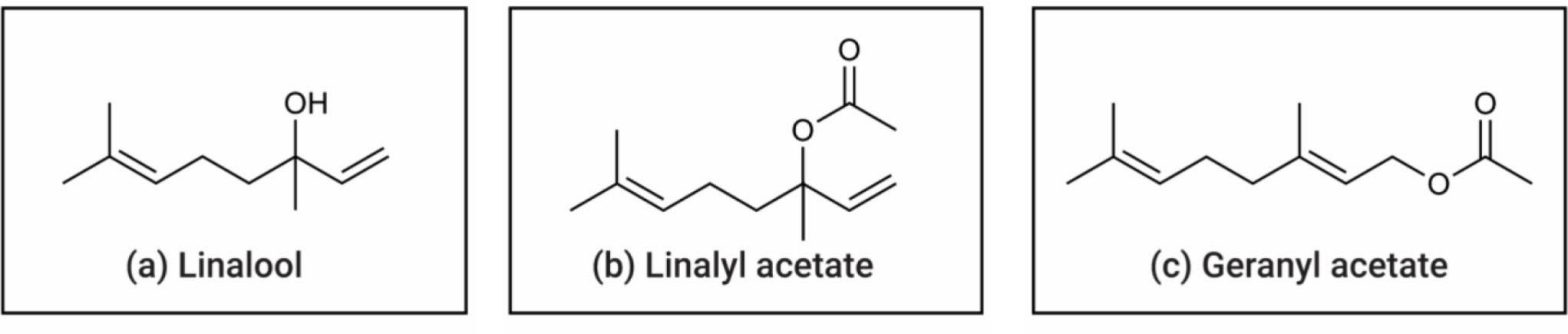
Chemical structures of linalool, linalyl acetate, and geranyl acetate

Plant-derived natural compounds, such as *Ginkgo biloba* [8], *Bacopa monnieri* [9], and *Coriandrum sativum* L [10], were found to be promising sources of drugs for neuroprotection due to their antioxidant property. Coriander (*Coriandrum sativum* L.), a plant belonged to *Apiaceae* family, is an edible plant with unique scent commonly found in Asia, Africa, and European countries. Coriander is not only used in culinary, but its essential oils are commonly applied as a base in perfumery and cosmetics [11, 12]. It is also found to be abundant in polyphenols, such as ferulic acid, caffeic acid, gallic acid, and chlorogenic acid [10]. Coriander is historically used in traditional medicines of many world regions as a medicinal herb to treat dietary problems, such as indigestion, nausea, and increasing appetite [13]. Of note, coriander exhibits antioxidant activity in both in vitro [14, 15] and in vivo studies [16]. Moreover, coriander enhances an anti-aging effect against UV-induced mice models *via* reducing ROS production and modulating the level of metalloproteinase-1 (MMP-1), a type of collagenase enzyme [17].

Linalool is the most abundant compound found in coriander, followed by linalyl acetate and geranyl acetate (Fig. 1). The linalool possesses various bioactivities, including neuroprotection. It was reported that the linalool minimized the DNA damage of the brain tissue in H_2_O_2_-induced mouse models [18]. The protective effect was also reported in the mice treated with twice a day linalool for a 3-month period, in which the reductions of several related markers were observed including a decreased level of β-amyloid, a protein related to AD pathology, as well as reduced levels of several proinflammatory markers (i.e., p38, MAPK, NOS2, COX-2, and IL-1β). This was suggested to be due to an anti-inflammatory effect of the compound [19]. Linalool is well-known for its effects on the central nervous system (CNS) and was noted to be a good candidate for several psychological disorders such as depression [18] and anxiety [20]. Linalool also exhibits an anxiolytic effect *via* γ-aminobutyric acid (GABA) receptor in mouse models [20]. Moreover, linalool was reported for its anticancer activity against several types of cancer cell lines such as leukemia [21], lymphoma [22], melanoma, and renal adenocarcinoma [23]. It was noted that the linalool induces apoptosis of the leukemic cells *via* upregulating expressions of p53 and acts as cyclin-dependent kinase inhibitors (CDKIs) [21]. Though geranyl acetate and linalyl acetate have not been directly addressed, it was found that the compounds, such as ursolic acid [24–26] and chlorogenic acid [27, 28], exhibited antioxidative, anti-inflammatory, and neuroprotective activities. Linalyl acetate is not only found in coriander, but also in other plants (i.e., lavender and kaffir lime). Studies revealed its neuroprotective activity through antioxidative activity *via* reducing ROS generation and LDH secretion in vitro [29] and in vivo studies of the ischemic injury model [30]. Linalyl acetate also displays neuroinflammatory activity *via* the suppression of TSLP/IL-33 signaling pathways, resulting in the decreases in proinflammatory cytokine secretion [31]. Linalyl acetate possesses anticancer [32] and pain-reliving [33] properties. The anticancer effect of linalyl acetate against the melanoma cells was noted to be by inducing ERK and JNK enzyme activities leading to apoptosis as well as by inhibiting ROS production [32]. Furthermore, it was shown that linalyl acetate and linalool act synergistically to potentiate anti-inflammatory effect of the essential oil [34]. Geranyl acetate is another type of acetate ester found in coriander as well as other plants (i.e., lemongrass, carrot, and neroli). Studies revealed the potential properties of geranyl acetate as a neuroprotective agent as the compound showed anti-inflammatory [35] and antioxidative properties [36] in vitro studies. Another study also revealed the neuroprotective activity of geranyl acetate that acts as an acetylcholinesterase (AChE) inhibitor [37]. It was found that the geranyl acetate at high concentrations exhibit anticancer activity against colon cancer *via* the downregulation of anti-apoptotic BCL-2 protein and upregulation of apoptotic BAX protein in the colon cancer cells [38].

Computational approaches have been employed as fundamental facilitating tools in drug development. Molecular docking is commonly used to elucidate the possible binding modalities of the interest compound against its target [39–41], which would be beneficial for further design to achieve new compounds with improved activity. Poor pharmacokinetics and drug toxicity are noted to be the main factors of failures in drug discovery, particularly in the late stage of the development pipeline. Therefore, the use of computational tools for predicting pharmacokinetics (i.e., absorption, distribution, metabolism, elimination, and toxicity; ADMET profile) of compounds at an early stage of development has gained considerable attention in order to decrease late-stage failure, increase success rate, and reduce time and cost [42]. It is also motivated to predict whether the compound has the potential to be administered as an oral drug [43]. Several rules were applied to determine the drug-likeness of the compounds, including Lipinski’s [44], Veber’s [45], and Egan’s [46] rules, in which the determination by each rule is based on different compounds’ properties.

Although linalool was reported as a neuroprotective agent in some literature, the studies regarding the neuroprotective potential of other two coriander-derived compounds (i.e., linalyl acetate and geranyl acetate) are still scarce. This study aimed to investigate the protective effects of three coriander-derived compounds (linalool, linalyl acetate, and geranyl acetate) against the OS-induced SH-SY5Y neuroblastoma cell line. Molecular docking was performed to elucidate possible targets, binding modalities, and key features essential for protein-ligand binding. In silico pharmacokinetics/toxicity profile was also conducted to predict drug-like properties and possibility for further development. The findings could be beneficial to reveal the knowledge that highlights the values of this edible plant as a candidate for the prevention or treatment of NDs.

## Materials and Methods

### Reagents and Chemicals

Human neuroblastoma SH-SY5Y cell line was obtained from the American Type Culture Collection (Manassas, VA, USA). Streptomycin and penicillin, fetal bovine serum (FBS), and Dulbecco’s modified Eagle’s Medium (DMEM) were obtained from Gibco BRL (Gaithersburg, MD, USA). Linalyl acetate was purchased from Acros Organics (Fairlawn, NJ, USA). 3-(4,5-Dimethylthiazol-2-yl)-2,5-diphenyl tetrazolium bromide (MTT), 2′,7′–dichlorodihydrofluorescin diacetate (H_2_DCFDA), rhodamine 123, trypsin-EDTA solution (0.25%), dimethyl sulfoxide (DMSO), linalool, geranyl acetate, and SIRT1 activity kit were obtained from Sigma-Aldrich (St. Louis, MO, USA). Hydrogen peroxide (H_2_O_2_, 30%) and Muse™ Annexin V & Dead Cell Assay Kit were obtained from Merck Millipore (Darmstadt, Germany). RIPA buffer was obtained from Cell Signaling Technology (Beverly, MA, USA).

### Cell Culture and Treatment

The SH-SY5Y cell line derived from neuroblastoma is commonly used as an in vitro model for studying neurotoxicity [47, 48]. In this study, SH-SY5Y cells were cultured with DMEM containing 10% FBS and 1% penicillin-streptomycin and maintained at 37 °C under humidified air with 5% CO_2_. The cultured media were refreshed every 2–3 days, and the cells were passaged when containing about 80% confluence. For cell treatment, SH-SY5Y cells at a concentration of 1.0 × 10^5^ cells/mL were cultured and then pretreated with linalool, linalyl acetate, and geranyl acetate at various concentrations for 3 h. The cells were incubated with 400 µM H_2_O_2_ for an additional 24 h. Cells without treatment were used as a control.

### Preparation of Tested Compounds

Linalool, linalyl acetate, and geranyl acetate were diluted to a concentration of 100 µM with DMSO into stock solution. Then the compounds were further diluted with DMEM supplemented with 10% FBS and 1% penicillin-streptomycin into various concentrations.

### Determination of Cell Viability by MTT Assay

MTT is a colorimetric assay used to determine cell metabolic activity as an indicator of cell viability [49]. Briefly, SH-SY5Y cells were cultured in 96-well plates and incubated overnight before treatment. Then MTT solution was added to each well and incubated at 37˚C for 3 h in the dark. After the incubation, crystal formazan was solubilized with 0.04 N HCl in isopropanol as an extraction buffer before measuring absorbance at 570 nm using a microplate reader (TECAN, Switzerland). The viability of the cells was analyzed as percentages related to the control group.

### Morphological Assessment of SH-SY5Y by Light Microscope

SH-SY5Y cells were cultured in cell culture dishes and treated as mentioned previously. Morphology of the treated and untreated cells was observed using an inverted light microscope (Olympus Corporation, Tokyo, Japan) with 20× magnification. Multiple images were taken for an individual treatment using a digital camera.

### Detection of Cell Apoptosis Using Flow Cytometry

Apoptotic profile was determined using specific fluorescent dyes such as a fluorescent-labeled annexin V to detect the translocation of phosphatidylserine, providing a detailed analysis of the early and late apoptotic population [50]. Briefly, 1.0 × 10^5^ cells/mL of SH-SY5Y cells were incubated overnight in 24-well plates. After the treatment, both adherent and floating cells were harvested and incubated with the fluorescent reagent. The percentages of living, apoptotic, and dead cells were analyzed using a Muse™ cell analyzer (Merck Millipore, Darmstadt, Germany).

### Measurement of ROS Production

Fluorescent H_2_DCFDA was used to determine intracellular ROS production to evaluate the OS in SH-SY5Y cells [51]. In brief, SH-SY5Y cells were seeded onto 96-well plates. After the treatment, cells were incubated with H_2_DCFDA at a final concentration of 10 µM for 30–45 min in the dark. Then, the emission wavelength at 528 nm and excitation wavelength at 485 nm were measured using a microplate reader.

### Determination of Mitochondrial Membrane Potential

Mitochondrial membrane potential (MMP) was assessed to understand the impact of the compounds on mitochondrial function [52]. In this study, rhodamine 123 was used to determine MMP. Briefly, SH-SY5Y cells were seeded onto 96-well plates. After the treatment, rhodamine 123 solution was added to each well and incubated in the dark for an additional 30 min at 37 °C under humidified air with 5% CO_2_. Then, emission spectra at 488 nm and excitation spectra at 525 nm were measured using a microplate reader.

### Determination of SIRT1 Deacetylase Activity

Sirtuin1 (SIRT1) deacetylase enzyme is involved in various cellular processes, including aging, metabolism, and stress response [53, 54]. The activity level of the enzyme was determined to explore the effect of the compounds on the regulation of this enzyme. SH-SY5Y cells were cultured in 6-well plates and treated with the compound for 3 h followed by H_2_O_2_ for another 24 h. The cells were rinsed with 1X PBS before incubating with 1X RIPA buffer containing protease inhibitor at 4˚C for 20 min. Harvested cells were then centrifuged at 12,000 rpm for 20 min, and the supernatant was collected. Concentrations of protein were determined using Bradford protein assay (Bio-Rad Laboratories, CA, USA). SIRT1 activity was measured according to the manufacturer’s protocols of the SIRT1 activity assay kit. Fluorescent intensity with excitation at 340 nm and emission at 445 nm was measured using a microplate reader. The activity of SIRT1 deacetylase was analyzed in percentages related to the control group.

### In Silico Pharmacokinetic Prediction

Insight into the ADMET profiles of the compounds was provided by in silico pharmacokinetic prediction. Various physicochemical properties of linalool, linalyl acetate, and geranyl acetate including molecular weight, number of rotatable bonds, number of hydrogen bond donors and acceptors, lipophilicity (logP), molar refractivity, topological polar surface area (TPSA), as well as the violations of Lipinski’s, Veber’s, and Egan’s rules were predicted using SwissADME, an online web tool created by Swiss Institute of Bioinformatics (http://www.swissadme.ch/) [55]. Lipophilicity was calculated from the arithmetic mean of five predictive models including XLOGP3, WLOGP, MLOGP, SILICOS-IT, and iLOGP. Pharmacokinetic properties including water solubility (logS), Caco2 permeability (logPapp in 10^− 6^ cm/s), intestinal absorption (%Absorbed), blood-brain barrier (BBB) permeability (logBB), CNS permeability (logPS), total clearance (log mL/min/kg), and cytochrome P450 substrate inhibition were calculated by pkCSM prediction tool (https://biosig.lab.uq.edu.au/pkcsm/) [56]. Toxicity was predicted using an online accessible web tool ProTox-II (https://tox-new.charite.de/protox_II/) [57]. The software classified the compounds into the Globally Harmonized System (GHS) toxicity classification ranged from I to VI, by its lethal dose of 50% (LD_50_). The compound with toxicity class I is the compound with the highest toxicity, while class VI is less likely to be toxic. The predicted toxicity parameters of the compound include hepatotoxicity, carcinogenicity, immunotoxicity, mutagenicity, cytotoxicity, and MMP.

### Computational Modeling

Molecular docking was performed to predict possible binding modes and ligand-protein interactions of geranyl acetate, linalyl acetate, and linalool against the activator binding site of SIRT1 deacetylase enzymes. Crystal structure of the human SIRT1 catalytic domain in complex with resveratrol molecules and acetylated p53 peptide conjugated with fluorophore (PDB ID: 5BTR) was used as a target protein for simulation. The protein structure was prepared by removing three resveratrol molecules and crystallographic water molecules, adding polar hydrogen atoms, and assigning Gasteiger atomic partial charges. Chemical structures of the compounds were obtained from PubChem and prepared by adding polar hydrogen atoms and Gasteiger atomic partial charges. Docking simulations were performed using AutoDockTools v.4.2.6 [58] in which rotational bonds of the compound structures were treated as flexible whereas those of the protein structures were regarded as rigid. Docking parameters were set with grid box size of 50 × 50 × 50 points and grid spacing of 0.375 Å. Searching area was defined to cover the interface between the catalytic and extended N-terminal domain of SIRT1, which is the binding site of the natural resveratrol. Initially, reliability of the docking protocol was validated by redocking of the co-crystalized ligand, and root mean squared standard deviation (RMSD) value was calculated by the difference between the original binding and redocking poses. Lamarckian genetic algorithm [59] with at least 1,000 runs for each compound was used as the search parameter. Other grid parameters and energy evaluations were left as defaults. Molecular interactions of each compound and SIRT1 were visualized and illustrated using Discovery Studio Visualizer v21 (BIOVIA, Dassault Systèmes).

### Statistical Analysis

All data are expressed as mean ± SD values of three independent experiments, which are defined by using distinct passages of SH-SY5Y cells. Statistical differences are determined by one-way analysis of variance (one-way ANOVA) and are presented with [F(DFn, DFp), p value]. Each data is compared using Tukey’s comparison test using GraphPad Prism 8 scientific software (GraphPad Software Inc., CA, USA). Probability value *p* < 0.05 is considered statistically significant.

## Results

### Coriander-derived Compounds Reduced Cytotoxicity of H_2_O_2_ on SH-SY5Y Cells

**Fig. 2:**
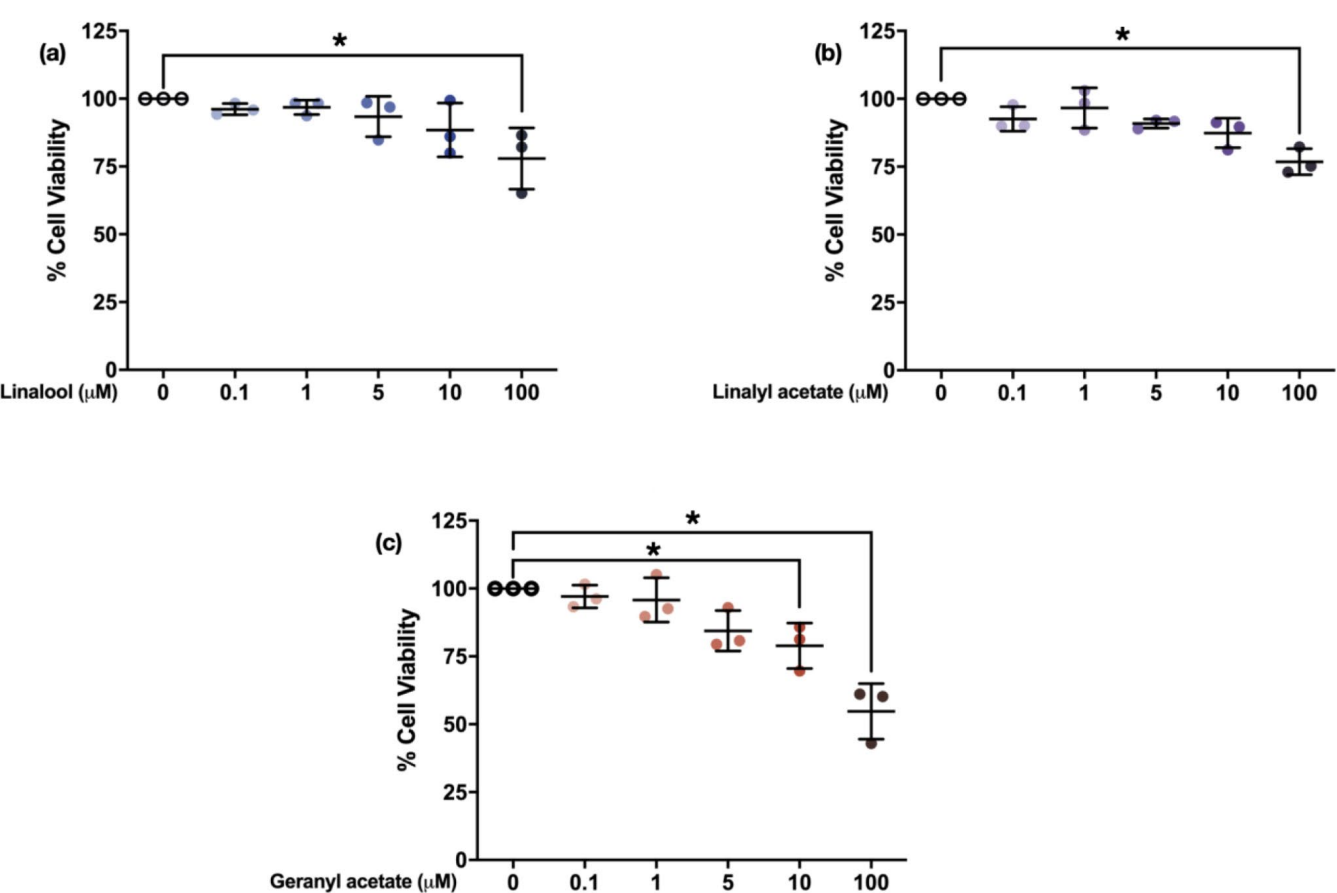
Scatter plot showing cytotoxicity of linalool, linalyl acetate, and geranyl acetate at various concentrations (0.1, 1, 5, 10, and 100 µM) on SH-SY5Y cells after treatment for 24 h (*N* = 3). *p ≤ 0.05 compared to the control group

**Fig. 3:**
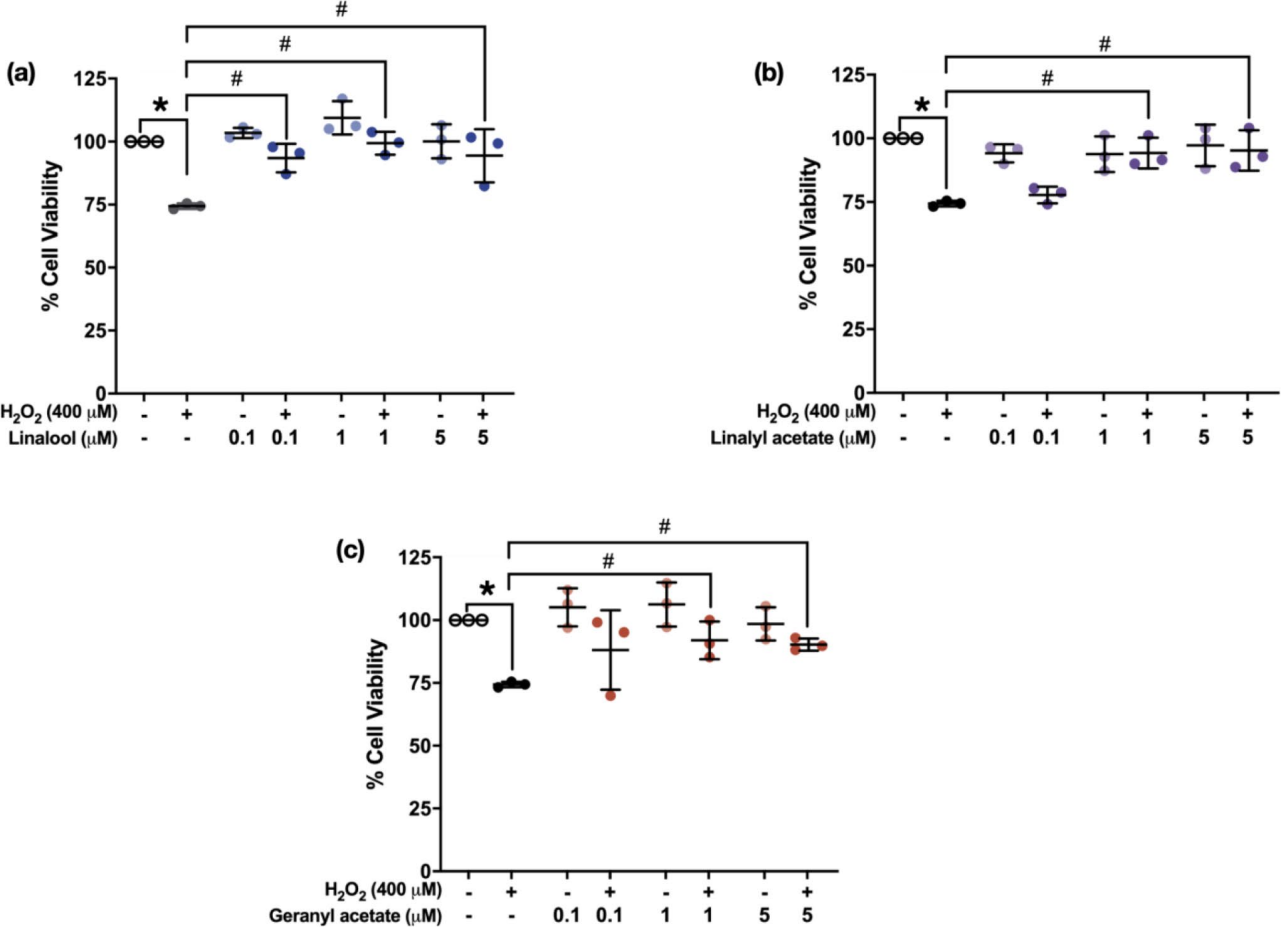
Scatter plot of neuroprotective effect of (**a**) linalool, (**b**) linalyl acetate, and (**c**) geranyl acetate at concentrations of 0.1, 1, and 5 µM (3 h) against viability of H_2_O_2_-induced SH-SY5Y cells (*N* = 3). *p ≤ 0.05 compared to the control group. #*p* ≤ 0.05 compared to the H_2_O_2_-induced group

**Fig. 4:**
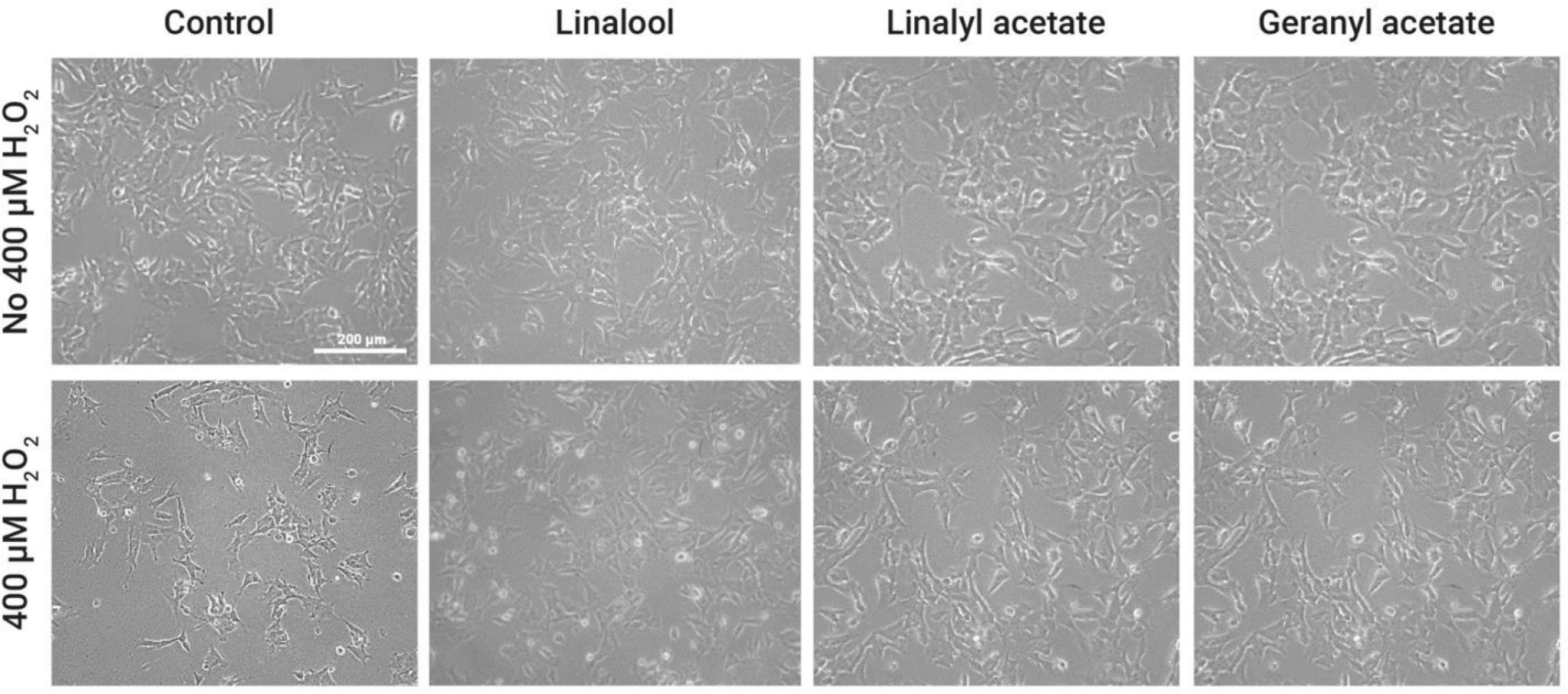
Cell morphology of H_2_O_2_-induced SH-SY5Y cells under the condition with or without 3 h pretreatment of linalool, linalyl acetate, and geranyl acetate at a concentration of 1 µM. Observations were made under inverted light microscope with 40X magnification, scale bar = 200 μm

Cytotoxicity of linalool, linalyl acetate, and geranyl acetate was determined using MTT assay by pretreating SH-SY5Y cells with various concentrations (0.1, 1, 5, 10, and 100 µM) of the tested compounds for 24 h. The results showed that linalool and linalyl acetate at a concentration of 100 µM (77.96 ± 11.28% and 76.83 ± 4.81%, respectively), and geranyl acetate at concentrations of 10 and 100 µM (78.91 ± 8.37% and 54.72 ± 10.23%, respectively) significantly decrease the viability of SH-SY5Y cells (Fig. 2). This suggests the possible toxicity at higher concentrations (10 and 100 µM). Accordingly, the compounds at concentrations of 0.1, 1, and 5 µM were selected for further study for their effects against the H_2_O_2_-induced SH-SY5Y cells. The cells were pretreated with linalool, linalyl acetate, and geranyl acetate for 3 h, before oxidative induction with H_2_O_2_ for another 24 h. Then, the cell viability was measured, and cell morphology was observed using the inverted microscope. The collected data were statistically compared to that of the H_2_O_2_-induced group (74.39 ± 1.12%). The results showed that pretreatments with 0.1, 1, and 5 µM of linalool (93.48 ± 5.64%, 99.35 ± 4.55%, and 94.42 ± 10.55%), as well as 1 and 5 µM of linalyl acetate (94.23 ± 6.05% and 95.23 ± 7.95%) and geranyl acetate (91.95 ± 7.47% and 90.28 ± 2.43%) can significantly increase the cell viability of the cells compared to the H_2_O_2_-induced cells [F [7, 16] = 9.937, *p* < 0.0001; F [7, 16] = 8.786, *p* = 0.0002; F [7, 16] = 5.343, *p* = 0.0027, respectively] (Fig. 3). The observed cell morphology showed that the H_2_O_2_-induced cells have lost their original morphology, while the cells that were pretreated with the three compounds have been revived into their normal morphologies (Fig. 4). Even though the higher concentration (100 µM) of the compounds was found to be toxic to the neuronal cell, the pretreatment of the compounds at lower concentrations (1–5 µM) presented a protective activity against OS-induced neurotoxicity in SH-SY5Y cell line.

### Coriander-derived Compounds Prevented Apoptotic Profiles Against H_2_O_2_-induced Cell Death

**Fig. 5:**
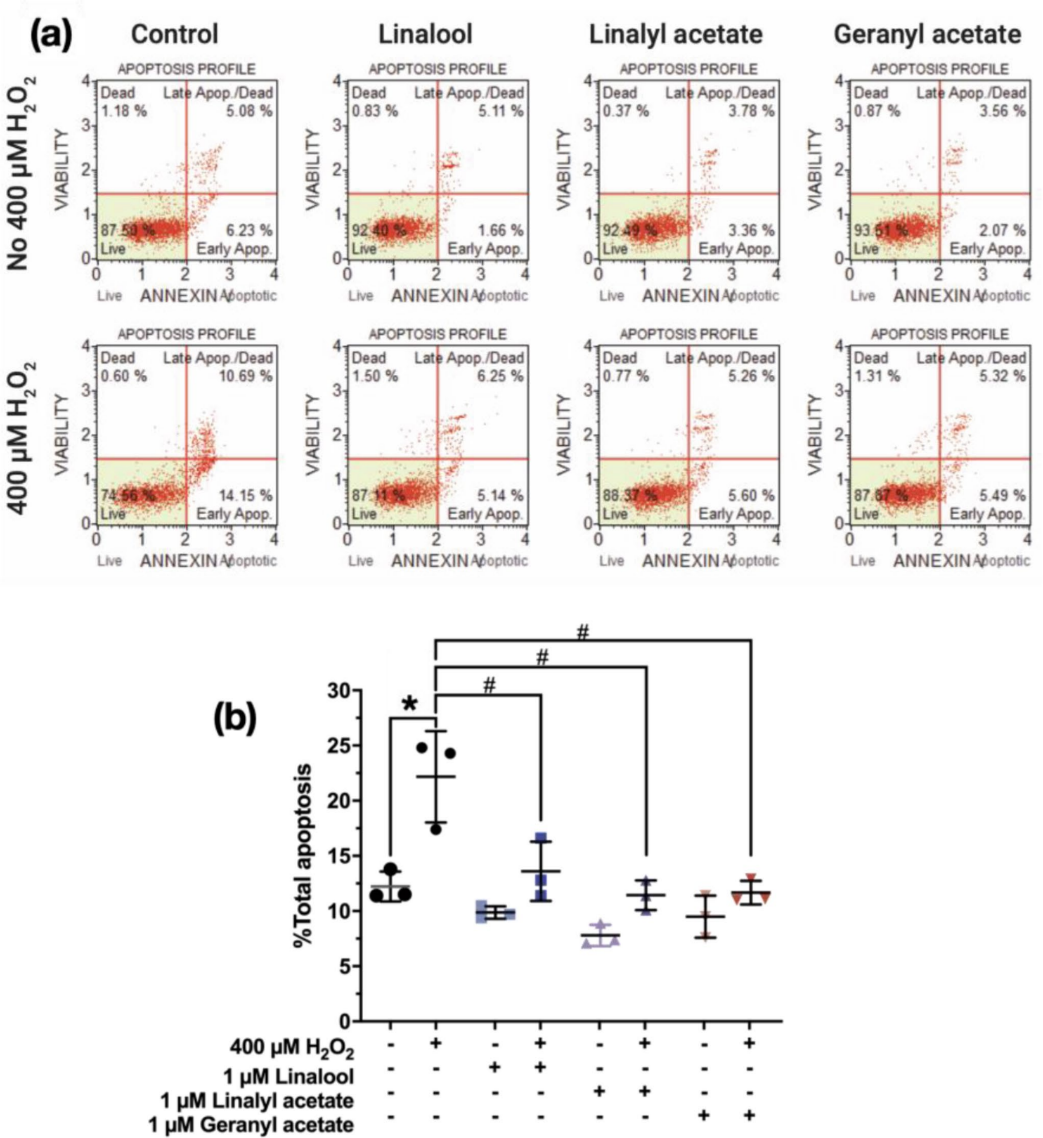
(**a**) Apoptotic profiles and (**b**) effect of pretreatment of linalool, linalyl acetate, and geranyl acetate (1 µM for 3 h) against H_2_O_2_-induced neurotoxicity (400 µM) (*N* = 3). *p ≤ 0.05 compared to the control group. #*p* ≤ 0.05 compared to the H_2_O_2_-induced group

To investigate effects of the studied compounds against apoptosis of the SH-SY5Y cells, cells were pretreated with 1 µM of each compound before treating with 400 µM of H_2_O_2_. The apoptosis of the cells was measured using flow cytometry then the data were statistically compared to the H_2_O_2_-induced cells. The results showed that the total apoptotic percentage of H_2_O_2_-induced cells (22.17 ± 4.14%) significantly increased when compared to the control group (12.23 ± 1.36%). It was found that linalool, linalyl acetate, and geranyl acetate alone showed no effect on inducing the apoptosis, but they can significantly prevent the apoptosis of the SH-SY5Y cells (as observed when compared to the H_2_O_2_-induced cells as the apoptotic percentage of 13.60 ± 2.69%, 11.43 ± 1.35%, and 11.67 ± 1.07%, respectively [F [7, 16] = 13.53, *p* < 0.0001] (Fig. 5). The results showed that pretreatment with linalool, linalyl acetate, and geranyl acetate (1 µM) prevented the apoptosis of OS-induced neuronal cell death.

### Decrease in ROS Production in Presence of Coriander-derived Compounds

**Fig. 6:**
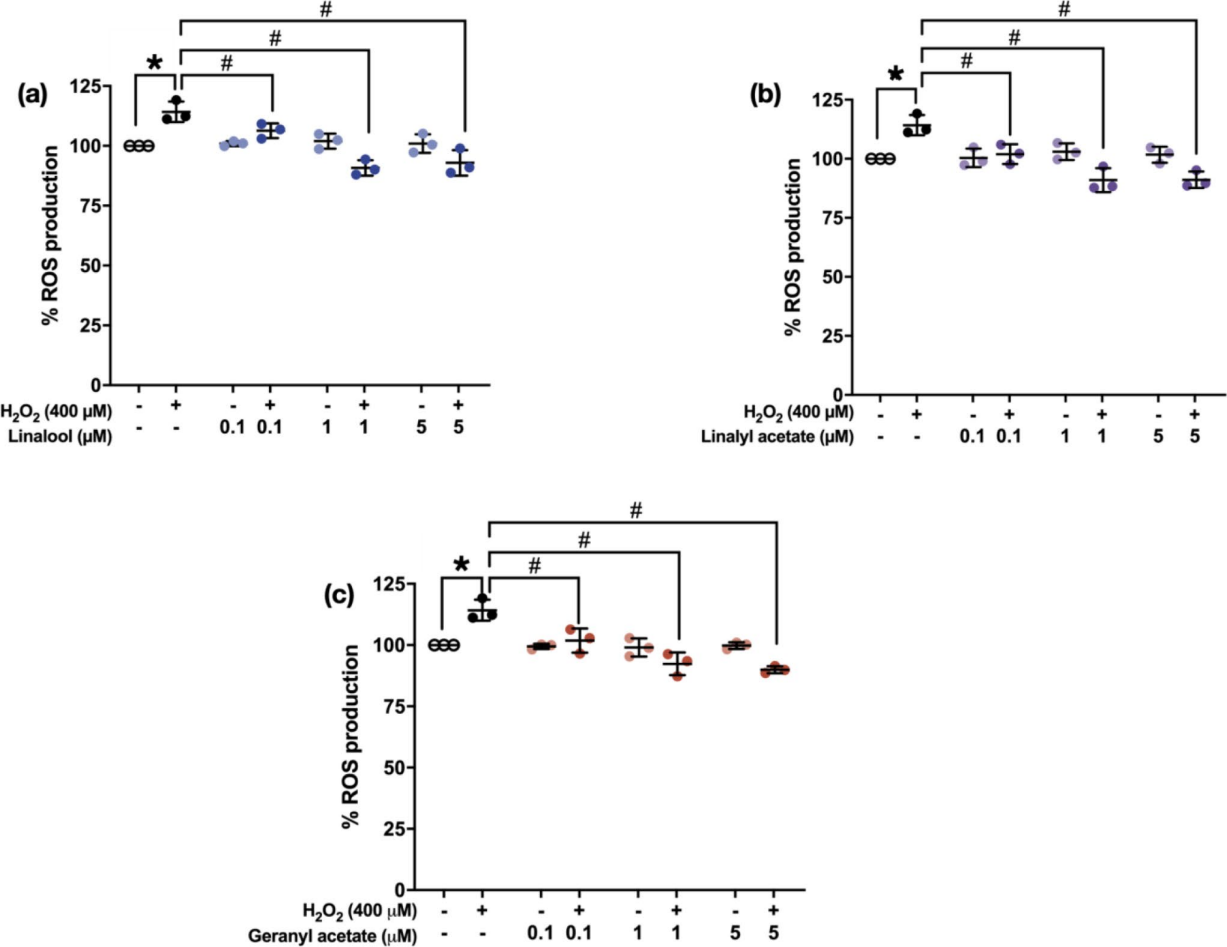
ROS production levels of the cells pretreated with (**a**) linalool, (**b**) linalyl acetate, and (**c**) geranyl acetate at concentrations of the 0.1, 1, and 5 µM (3 h) against H_2_O_2_-induced SH-SY5Y cells (400 µM) (*N* = 3). *p ≤ 0.05 compared to the control group. #*p* ≤ 0.05 compared to the H_2_O_2_-induced group

To investigate the ROS production of the cells, SH-SY5Y cells were pretreated with 0.1, 1, and 5 µM of the tested compounds for 3 h before treating with 400 µM of H_2_O_2_ for another 24 h. ROS productions were determined by DCFDA assay. The results were statistically compared to the H_2_O_2_-treated cells (114.2 ± 4.30%) which significantly increased intracellular ROS production compared to the control group (100%). While pretreatments with 0.1, 1, and 5 µM of linalool (106.3 ± 3.12%, 90.80 ± 3.24%, and 92.91 ± 5.351%), linalyl acetate (102.0 ± 4.162%, 90.95 ± 5.05%, and 91.12 ± 3.52%), and geranyl acetate (101.9 ± 4.96%, 92.33 ± 4.63%, and 89.97 ± 1.41%) significantly reduced the ROS production when compared to the H_2_O_2_-induced SH-SY5Y cells [F [7, 16] = 13.75, *p* < 0.0001; [F [7, 16] = 11.43, *p* < 0.0001; F [7, 16] = 15.03, *p* < 0.0001] (Fig. 6). This finding suggested the antioxidative properties of the three coriander-derived compounds reduced excessive ROS in the oxidative damage.

### Coriander-derived Compounds Maintained MMP on H_2_O_2_-induced SH-SY5Y Cells

**Fig. 7:**
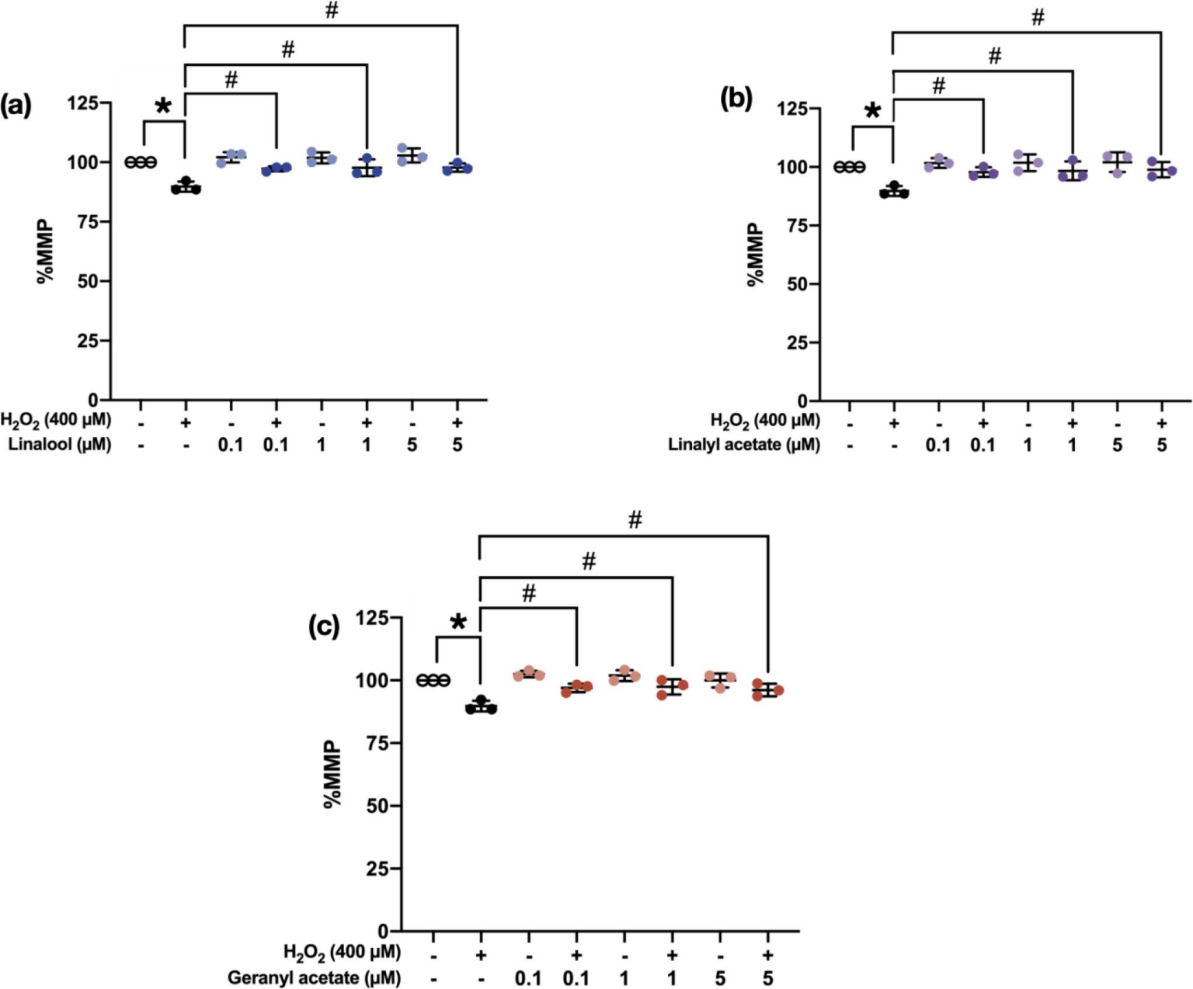
Scatter plot showing the MMP percentage of the cells pretreated with (**a**) linalool, (**b**) linalyl acetate, and (**c**) geranyl acetate at concentrations of 0.1, 1, and 5 µM (3 h) against H_2_O_2_-induced neurotoxicity (400 µM) (*N* = 3). *p ≤ 0.05 compared to the control group. #*p* ≤ 0.05 compared to the H_2_O_2_-induced group

MMP indicating mitochondrial functions was determined by Rhodamine123 staining. SH-SY5Y cells were treated with 0.1, 1, and 5 µM of the tested compounds for 3 h, then treated with 400 µM of H_2_O_2_ for another 24 h, followed by MMP determination. The data were statistically compared to the H_2_O_2_-treated cells (89.77 ± 2.11%). The following results showed that pretreatments with 0.1, 1, and 5 µM of linalool (97.26 ± 1.07%, 97.69 ± 3.51%, and 97.80 ± 1.77%, respectively), linalyl acetate (97.80 ± 2.07%, 98.35 ± 4.04%, and 98.87 ± 3.25%, respectively), and geranyl acetate (96.99 ± 1.695%, 97.41 ± 3.06%, and 96.15 ± 2.53%, respectively) significantly recovered the MMP of SH-SY5Y cells compared to the H_2_O_2_-induced cells [F [7, 16] = 10.69, *p* < 0.0001; F [7, 16] = 5.495, *p* = 0.0023; F [7, 16] = 10.74, *p* < 0.0001; respectively] (Fig. 7). Thus, the pretreatment of coriander-derived compounds protected mitochondrial dysfunction of the OS-induced SH-SY5Y neuronal cells.

### Coriander-derived Compounds Promoted SIRT1 Deacetylase Activity in H_2_O_2_-Insulted Neurons

**Fig. 8:**
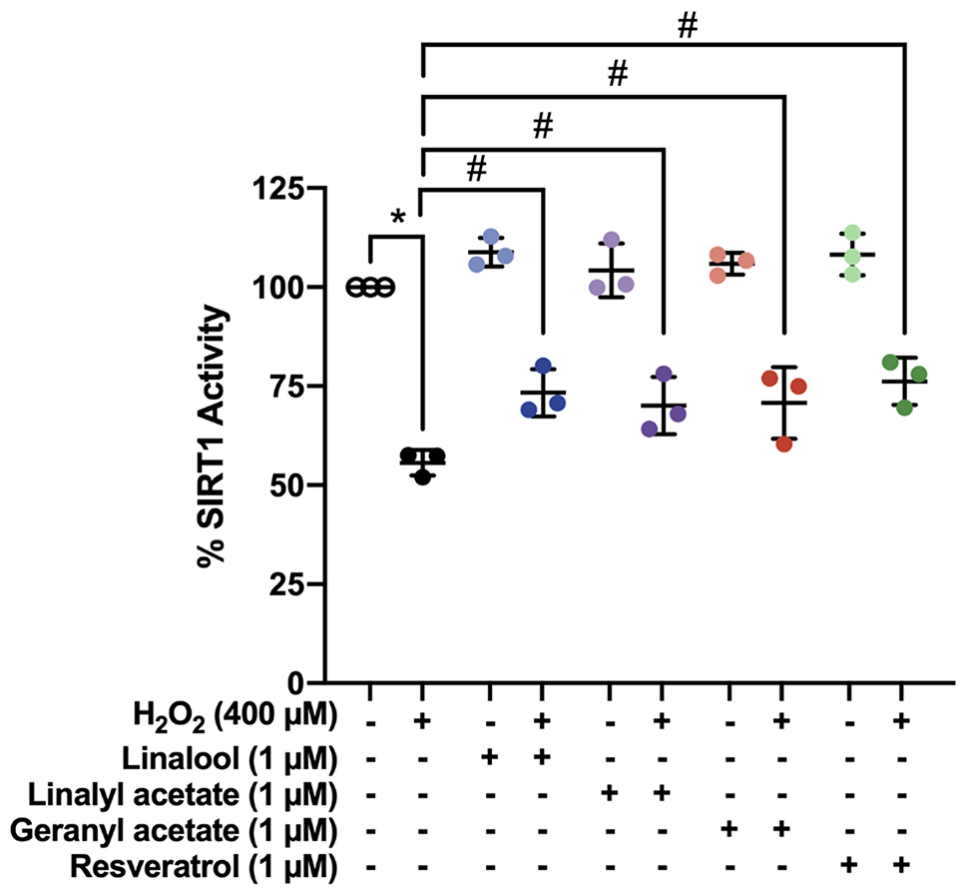
Scatter plot showing SIRT1 activity percentage of pretreatment of linalool, linalyl acetate, geranyl acetate, and resveratrol (1 µM for 3 h) against H_2_O_2_-induced SH-SY5Y cells (400 µM) (*N* = 3). *p ≤ 0.05 compared to the control group. #*p* ≤ 0.05 compared to the H_2_O_2_-induced group

SIRT1 deacetylase activity in SH-SY5Y cells pretreated with linalool, linalyl acetate, and geranyl acetate was determined. The H_2_O_2_-induced SH-SY5Y neuronal cells (55.66 ± 3.19%) showed a significant decrease in SIRT1 activity when compared to the control. While the cells pretreated with linalool (73.31 ± 5.96%), linalyl acetate (70.09 ± 7.22%), and geranyl acetate (70.76 ± 9.07%) displayed a significant increase in SIRT1 activity when compared to the H_2_O_2_-induced group [F [9, 20] = 38.53, *p* < 0.0001]. Moreover, the SIRT1 activating effects of the compounds were comparable to that of the well-known SIRT1 activator, resveratrol, which provided the SIRT1 activity percentage of 76.19 ± 5.96%. (Fig. 8). The results indicated that these three coriander-derived compounds acted as SIRT1 activators and could play a major role in SIRT1 modulating several downstream biological processes.

### In Silico Pharmacokinetics and Toxicity Predictions of Coriander-derived Compounds

**Fig. 9:**
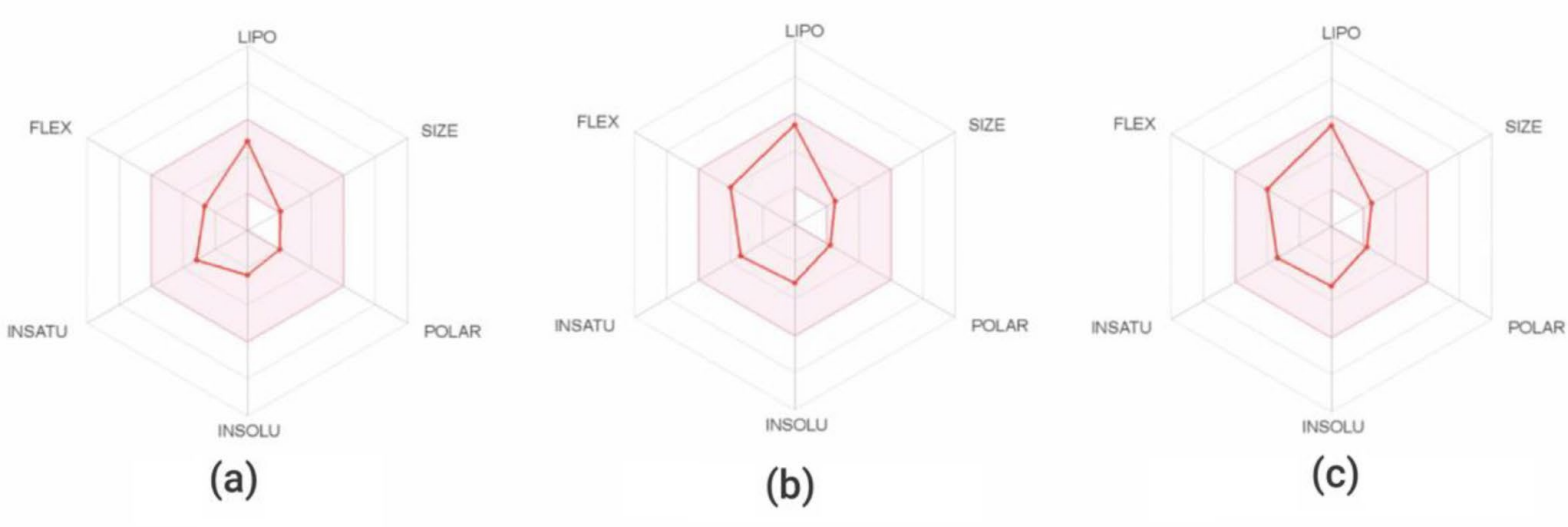
Bioavailability radar diagrams of (**a**) linalool, (**b**) linalyl acetate, and (**c**) geranyl acetate visualized through their physicochemical properties including lipophilicity (LIPO), size, polarity (POLAR), solubility (INSOLU), saturation (INSATU), and flexibility (FLEX). Shaded area indicated the optimal range, while red dots and lines indicated the compound properties

Drug-likeness of the linalool, linalyl acetate, and geranyl acetate was determined to consider the possibility of the compounds to be further developed as drugs. Drug-likeness of the studied compounds was predicted using SwissADME and verified based on Lipinski’s rule of five, Veber’s rule, and Egan’s rule. According to the Lipinski’s rule, the compound is considered drug-like if it has a molecular mass less than 500 g/mol, high lipophilicity (logP ≤ 5), ≤ 5 hydrogen bond donors, and ≤ 10 hydrogen bond acceptors [44]. In Veber’s rule, the compound is considered a drug-like agent if it has a TPSA less than 140 and a rotatable bond less than 10 [45]. Egan’s rule states a drug-like compound with good bioavailability with TPSA between 0 and 132 and lipophilicity between − 1 and 6 [46]. It was found that the calculated values of all investigated parameters (i.e., molecular weight, rotatable bonds, hydrogen bond donor, hydrogen bond acceptor, TPSA, and logP) of the linalool were lower than those of others (Table 1). The molecular weight of linalool, linalyl acetate, and geranyl acetate was less than 500 g/mol (MW: linalool = 154.25, linalyl acetate = 196.29, and geranyl acetate = 196.29 g/mol). All compounds displayed high lipophilicity providing logP values less than 5 (logP values: linalool = 2.6698, linalyl acetate = 3.2406, and geranyl acetate = 3.2422). TPSA of the compounds was between 20.23 and 26.30 Å². The results indicated that these three coriander-derived compounds complied with all conditions of Lipinski’s rule as well as satisfied both Veber’s and Egan’s rules (Table 1). Furthermore, bioavailability radar diagrams of linalool, linalyl acetate, and geranyl acetate (Fig. 9a-c) revealed that all six investigated physiochemical properties of these compounds fell entirely into the shaded area of the radar suggesting their drug-likeness.

**Table 1:** Physicochemical properties of linalool, linalyl acetate, and geranyl acetate

	Drug-likeness (Rules)					Linalool	Linalyl acetate	Geranyl acetate
	Lipinski’s		Veber’s		Egan’s			
**Molecular weight (g/mol)**	≤ 500	-		-		154.25	196.29	196.29
**Rotatable bonds**	-	≤ 10		-		4	6	6
**H-bond acceptors**	≤ 10	-		-		1	2	2
**H-bond donors**	≤ 5	-		-		1	0	0
**LogP**	≤ 5	-		-1–6		2.6698	3.2406	3.2422
**TPSA (Å²)**	-	≤ 140		0–132		20.23	26.30	26.30

**Fig. 10:**
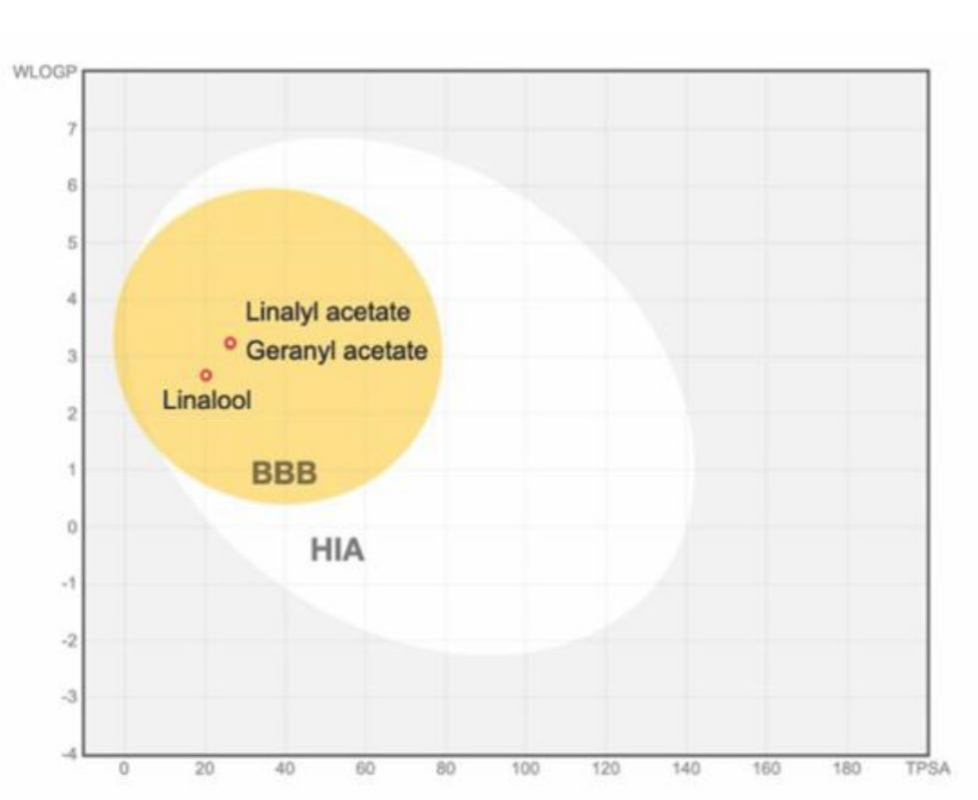
BOILED-Egg plot illustrated high probabilities of passive gastrointestinal absorption (white) and blood-brain penetration (yellow) of linalool, linalyl acetate, and geranyl acetate

Pharmacokinetics and toxicity (ADMET) profile of the compounds were further investigated using pkCSM software. Absorption ability of the compounds is considered by its water solubility (logS), Caco2 permeability (logPapp in 10^− 6^ cm/s), and intestinal absorption. Water solubility of the compounds (logS) indicates the solubility of compounds in 25˚C water, in which the value is expressed as logarithm of the molar concentration (mol/L). According to solubility criteria, the solutes that require more than 10,000 parts per part of solvent are considered insoluble [60]. The results showed that linalool, linalyl acetate, and geranyl acetate displayed logS values of -2.292, -2.942, and − 3.326, respectively. This suggested that all three compounds are water-soluble. It was suggested the compounds with calculated logPapp value > 0.9 as compounds to high Caco2 permeability [56]. Similarly, the compounds with %Absorbed > 30% are noted to be highly intestinal absorbed [56]. Accordingly, the prediction results showed that all studied compounds provide high Caco2 permeability (logPapp: linalool = 1.405, linalyl acetate = 1.61, and geranyl acetate = 1.643) as well as the high percentage of intestinal absorption (%Absorbed: geranyl acetate = 93.649%, linalyl acetate = 95.299%, and geranyl acetate = 95.579%). To be used as neuroprotective agents, the ability of the compound to pass across the BBB to reach its target site in the brain is highly concerned. This distribution ability was determined by BBB permeability (logBB) and CNS permeability (logPS). Compounds with values of logBB > 0.3 and logPS > -2.0 are determined to readily pass across into the brain [56]. Accordingly, all compounds have high possibility to pass across the BBB with moderate distribution ability into the CNS (logBB: linalool = 0.608, linalyl acetate = 0.527, and geranyl acetate = 0.581, logPS: linalool = -2.28, linalyl acetate = -2.383, and geranyl acetate = -2.219, Table 2). The results were also supported by the BOILED-Egg plot which suggested that all compounds have a high probability of absorption through the gastrointestinal tract [55] as well as passing across the BBB (Fig. 10). Potential of each compound to act as an inhibitor of cytochrome P450 (CYP450) isozymes, a major class metabolizing enzyme played a role in the metabolism of a wide range of drugs, was determined regarding the drug-drug interaction issue [61]. It was indicated that linalool, linalyl acetate, and geranyl acetate were not inhibitors of investigated CYP450 isozymes, which suggested their low probability to cause drug-drug interaction (Table 2).

**Table 2:** Prediction of pharmacokinetic properties of coriander-derived compounds

	Linalool	Linalyl acetate	Geranyl acetate
***Absorption***			
Water solubility (log mol/L)	-2.292	-2.942	-3.326
Caco2 permeability (logPapp in 10^− 6^ cm/s)	1.405	1.61	1.643
Intestinal absorption (human) (% Absorbed)	93.649	95.299	95.579
***Distribution***			
BBB permeability (logBB)	0.608	0.527	0.581
CNS permeability (logPS)	-2.28	-2.383	-2.219
***Metabolism***			
CYP1A2 inhibitor	No	No	No
CYP2C19 inhibitor	No	No	No
CYP2C9 inhibitor	No	No	No
CYP2D6 inhibitor	No	No	No
CYP3A4 inhibitor	No	No	No

Furthermore, toxicity profile of linalool, linalyl acetate, and geranyl acetate were determined using ProTox-II software. LD_50_ values of linalool, linalyl acetate, and geranyl acetate are 2,200, 12,000, and 5,000 mg/kg, respectively. Linalool and geranyl acetate are classified as GHS toxicity class V, while linalyl acetate is categorized as GHS toxicity class VI. This suggested that all compounds are considered mildly toxic. It was also found that all three compounds display no hepatotoxicity, carcinogenicity, immunotoxicity, mutagenicity, and cytotoxicity. Moreover, these compounds are non-harmful to the MMP of the cells (Table 3).

**Table 3:** Prediction of toxicity of coriander-derived compounds

	Linalool	Linalyl acetate	Geranyl acetate
Toxicity class	V	VI	V
LD_50_ (mg/kg)	2,200	12,000	5,000
Hepatotoxicity	No	No	No
Carcinogenicity	No	No	No
Immunotoxicity	No	No	No
Mutagenicity	No	No	No
Cytotoxicity	No	No	No
MMP	No	No	No

### Binding Interaction of Coriander-derived Compounds on SIRT1

**Fig. 11:**
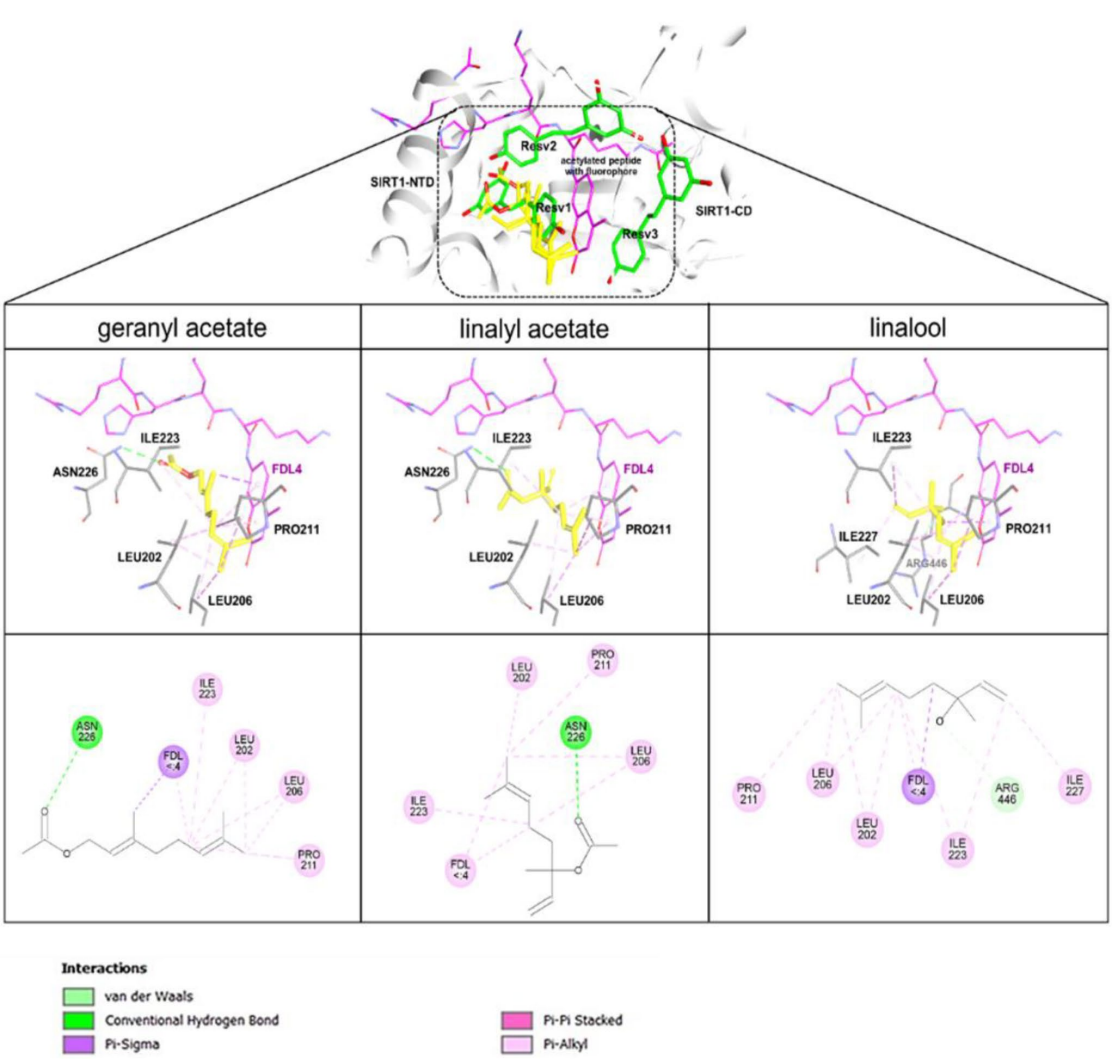
Molecular docking of geranyl acetate, linalyl acetate, and linalool on SIRT1 (PDB ID: 5BTR). 3D illustrations reveal the docked conformation of compounds on the activator binding site of the target enzyme. All three compounds (carbon in yellow), fluorogenic acetylated peptide (carbon in pink), interacting residues (carbon in grey), and resveratrol (carbon in green) are shown in a stick model. 2D illustrations reveal molecular interactions of the compounds (grey line) with binding site amino acid residues (colored ball). Polar and non-polar interactions are indicated in green and pink dotted lines, respectively. NTD, N-terminal domain; CD, C-terminal domain of SIRT1; FDL, coumarin moiety of fluorogenic peptide.

Molecular docking was performed to reveal possible binding modes and binding behaviors of geranyl acetate, linalyl acetate, and linalool against the target protein SIRT1. The simulation was initially validated by the redocking of the natural ligand, resveratrol, onto the SIRT1 binding site. The redocked poses of resveratrol molecules on SIRT1 were closely located to those originally observed in the crystal structure providing RMSD less than 2 Å (Fig. S1) [62], suggesting the reliability of the docking protocol. Docking simulations demonstrated that all coriander-derived compounds could accommodate within the binding site of resveratrol molecule 1 (Fig. 11, **upper**). The estimated binding free energy for the docking poses of geranyl acetate, linalyl acetate, and linalool were − 6.55, − 6.28, and − 5.36 kcal/mol, respectively, while that of the resveratrol was about − 7.68 kcal/mol. Ligand-protein interaction analyses revealed that all compounds interacted with several amino acid residues located on the N-terminal domain and the fluorophore-attached peptide (Fig. 11, **middle**). These interactions involved nonpolar contacts of LEU202, LEU206, PRO211, ILE223, and coumarin moiety of fluorogenic peptide with the aliphatic chain of the compounds, as well as by hydrogen bonds formed between ASN226 and acetate group of the compounds (Fig. 11, **lower**).

## Discussion

Neuronal cell death is a key harmful event of NDs, primarily caused by an imbalance between ROS production and antioxidant defense of the cells. Increased levels of extracellular ROS, such as H_2_O_2_, subsequently increase of intracellular free radicals and OS [63]. This study revealed that cells exposed to extracellular H_2_O_2_ showed increased intracellular ROS production. The intracellular OS can activate several damages to the cellular structure (including membranes, organelles, and genetic structures) which will lead to cellular malfunction and cellular death [5]. This supported the finding which showed a decrease in viability and MMP, as well as an increase in cellular apoptosis of the SH-SY5Y neuronal cells exposed to H_2_O_2_. Irreversible and progressive loss of specific neuronal cells promotes the progression of the NDs, in which effective treatments are currently unavailable [64]. Discovering a potential therapeutic and preventive agent for the early stage of NDs is crucial. Natural products, such as coriander, are well-known for their high structural diversity and have gained considerable attention as a potential alternative source for discovery of bioactive agents for treatment and prevention of NDs.

Coriander is an edible plant which is accessible and highly engaged in our daily life. Coriander was reported for its AD benefits. Ethanol extract of coriander leaves has been shown to revive the decrease in cell viability against amyloid beta (Aβ)-induced human neuroblastoma SH-SY5Y cell death as well as in the primary culture mouse cortical neurons [65]. Coriander also possesses strong antioxidant activity which can reverse Aβ-induced oxidative effects by increasing glutathione peroxidase (GPX) and catalase levels, while decreasing SOD levels [66, 67]. Additionally, inhalation of coriander oil decreased the Aβ deposition and OS markers (such as LDH and MDA) in the hypothalamus of Aβ-injected mice, improving the memory and depression behaviors of the AD mice [66]. In addition to OS, neuroinflammation significantly contributes to ND progression [68]. Though neuroinflammation is an innate defense line against pathogens in the brain, excessive neuroinflammation can induce neurotoxicity leading to neuronal cell death [68]. Previous studies suggested coriander as a neuroprotective candidate partly due to its anti-inflammatory properties [66].

Essential oil from the coriander, enriched with polyphenols and terpenes, particularly linalool, linalyl acetate, and geranyl acetate, which was investigated in this study. Pretreatment with these compounds significantly restored the cell viability against OS-induced SH-SY5Y neuronal cells. Increase in cell viability may result from several aspects, such as increase in cell longevity and cell proliferation, or decrease in cell death. Apoptosis is one of the cell death mechanisms playing a crucial role in neurodegeneration. The inner cell membrane, phosphatidylserine, will migrate through the surface of the cell during apoptosis, allowing its binding with annexin V. Our study showed that the pretreatment of three coriander-derived compounds reduced the apoptotic rate of the OS-induced neuronal cells. This aligns with another study which reported that linalool can reduce the level of cell cycle arrest in H_2_O_2_-induced PC-12 cells [69]. An increase in cell survival and decrease in cell death is found to be related to the ability of the compounds to mitigate intracellular accumulation of ROS and mitochondrial dysfunction. Mitochondria, a cellular organelle essential for cell survival, undergo morphological changes or dysfunction that accelerate the processes of disease pathogenesis, such as increasing the formation of Aβ proteins in AD or increasing the production of α-synuclein protein in PD [70]. High levels of glutamate, a well-known excitatory neurotransmitter, can lead to mitochondrial morphological change and dysfunction. However, a previous study indicated that this glutamate-induced OS and excitotoxicity condition were restored *via* the reductions of mitochondrial OS and calcium levels in linalool-treated HT-22 cells [71], which was concurrent with this study of restoring the MMP and reducing the excessive ROS levels against OS-induced SH-SY5Y cells by linalool, linalyl acetate, and geranyl acetate pretreatments. Moreover, linalool exerted protection against excitotoxicity in N-methyl-D-aspartate receptor of Drosophila AD model *via* decreasing the ROS accumulation and neuroinflammation as well as increasing the survival rate [72]. Similarly, oral administration of linalool in the AD mice model showed a significant decrease in inflammatory response [19]. Linalool-treated mice also revealed a significant decrease in AD pathology hallmarks including Aβ and tau protein aggregation, which leads to the improvements in its spatial learning and memory skills [19]. Unlike linalool, the neuroprotective effects of linalyl acetate and geranyl acetate are scarcely reported. However, both compounds can act as antioxidants, which is a key property for their related activities such as anticancer [32, 39], anti-inflammatory [34], and cardioprotective activities [73].

SIRT1 is a deacetylase enzyme generally expressed in the nucleus of neuronal cells. Many studies showed that the increase in expression and activation of SIRT1 deacetylase activity plays important roles in neuroprotection through its functionality modulating the downstream targets of anti-inflammatory, cellular metabolic, and antioxidant activities [74, 75]. This study showed that linalool, linalyl acetate, and geranyl acetate effectively stimulated intracellular SIRT1 activity in the OS-induced SH-SY5Y neuronal cells, with comparable efficacy to that of the resveratrol, a well-known SIRT1 activator [76]. The molecular docking results showed that geranyl acetate, linalyl acetate, and linalool could occupy within the same accommodated site of the resveratrol molecule 1 (Resv1) and interact with the key amino acid residues on the NTD of SIRT1 (i.e., LEU202, LEU206, ILE223, and ASN226) as well as with the coumarin moiety of fluorogenic peptide. Notably, the simulation demonstrated that geranyl acetate and linalyl acetate could form hydrogen bonding with the ASN226 residue of the SIRT1 through their carbonyl group, whereas this interaction was absent for that of the linalool whose chemical structure lacks the carbonyl group. This suggested that the carbonyl group presented in the molecule is essential for the formation of hydrogen bond interaction with the ASN226. The ASN226 residue was documented as one of the key residues for the binding of resveratrol against the SIRT1 due to its lower K_m_ value for substrate binding as well as its role in deacetylation rate of SIRT1 [62]. Previous molecular dynamics studies demonstrated that the binding of the Resv1 is stable to promote a tight interaction between acetylated fluorogenic peptide and SIRT1 catalytic domain [77]. These recent molecular docking studies supported the promising SIRT1 activators of coriander-derived compounds through the possible binding interaction and shared key amino acid residues of resveratrol in the SIRT1 active binding site, similar to our previous studies of plant-based polyphenolics and nitroxoline could occupy the same binding site of the Resv1 [78, 79]. Taken together, it is suggested that three coriander-derived compounds could mimic the binding behavior of the Resv1 and act as SIRT1 activators.

Three coriander-derived compounds were predicted for their pharmacokinetic properties and toxicity using computational tools to ensure their possibility of further drug development as effective therapeutics with minimized toxicities. Oral administration is considered the most effective, non-invasive, and convenient route of drug administration, which requires drug-likeness, drug solubility, as well as mucosal permeability as key properties [32]. Results showed that all compounds comply with three drug-likeness rules (i.e., Lipinski’s, Veber’s, and Egan’s rules). Moreover, all compounds were predicted to be soluble at room temperature as well as exhibit high permeability through Caco2 cells and high percentage of intestinal absorption. Furthermore, it was revealed that these coriander-derived compounds can pass across the BBB to reach target site of their actions in the CNS. Taken together, it is suggested that all compounds demonstrate high possibility to be further developed as oral drugs for neurodegenerative treatment. Drug-drug and food-drug interaction are issues to be concerned for effective treatment and patient safety, which can occur when more than one drug, or food and drug are co-administered. This event could lead to an alteration of the CYP450 enzymatic function (either by inhibition, competition, or induction) which affects the rate of drug metabolism and concentration of drug in active form leading to ineffective/impaired pharmacological efficiency or toxicity due to the accumulation of the drug [80, 81]. Herein, three compounds were predicted for their possibilities to act as inhibitors against the major classes of CYP450s with clinical importance (i.e., CYP1A2, CYP2C19, CYP2C9, CYP2D6, and CYP3A4). The predictions indicated that linalool, linalyl acetate, and geranyl acetate are not inhibitors of these metabolizing enzymes, suggesting their low possibilities of causing drug-drug interaction. This study highlights the potential of coriander-derived compounds, particularly linalool, linalyl acetate, and geranyl acetate, as neuroprotective agents against OS-induced neuronal damage. The in vitro analyses are supported by computational prediction to evaluate the compound’s potential drug-like ability and strengthen this research due to its dual approaches. This research extends previous work on natural compound’s neuroprotective effects, suggesting significant implications for developing novel and effective treatments for NDs and highlighting the importance of early intervention in disease progression. However, further studies to explore the detailed mechanisms of action of these bioactive compounds, in vivo studies, and clinical trials are necessary to fully establish their efficacy and safety for therapeutic use in NDs.

## Conclusion

**Fig. 12:**
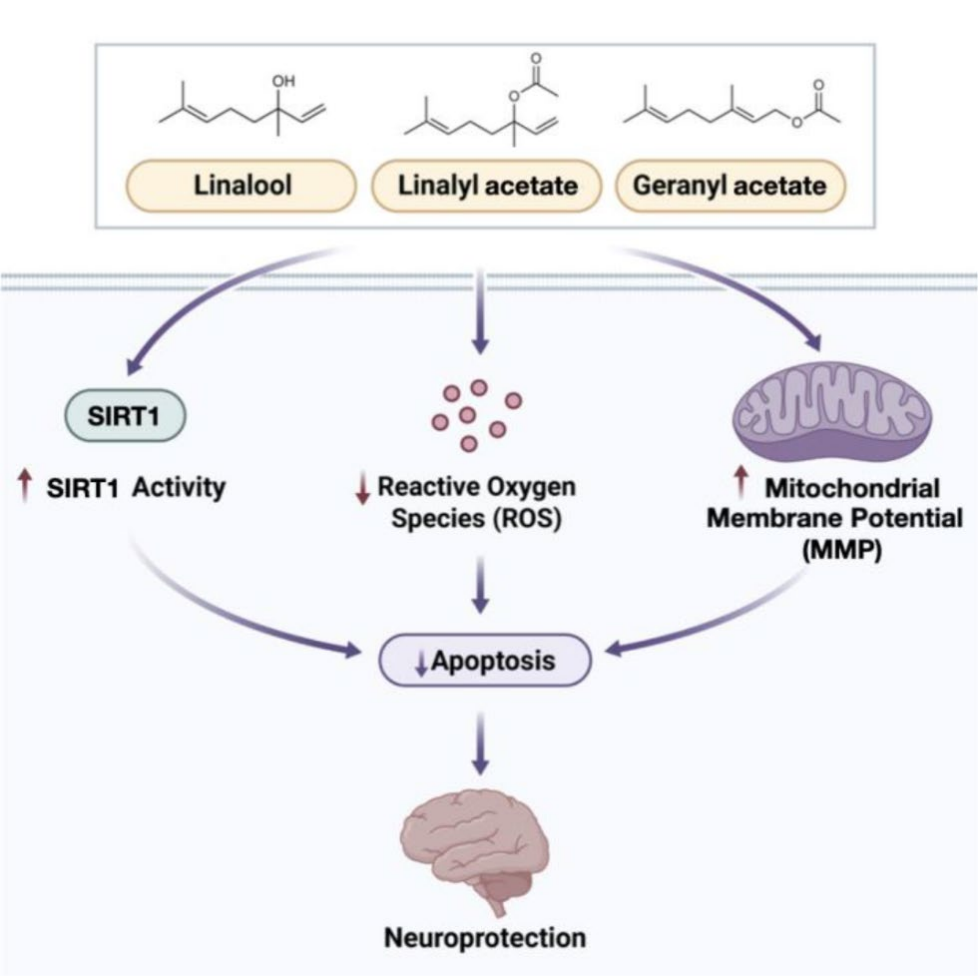
Possible neuroprotective activity of linalool, linalyl acetate, and geranyl acetate against the OS-induced neuronal cells. All three coriander-derived compounds prevented the accumulation of intracellular ROS, improved MMP, modulated SIRT1 enzyme activity, and reduced cellular apoptosis of the SH-SY5Y cells

Discovery of neuroprotective agents to slow down the progression and prevention of NDs is essential in the era of aging society. This study demonstrated that three coriander-derived compounds (i.e., linalool, linalyl acetate, and geranyl acetate) exhibited satisfying protective effects against the OS-induced cell death in SH-SY5Y neuronal cells *via* decreasing intracellular ROS levels, improving mitochondrial function, and stimulating SIRT1 activity (Fig. 12). Both in vitro and in silico molecular docking results supported that linalool, linalyl acetate, and geranyl acetate could act as SIRT1 activators and mimic the binding modality of the well-known SIRT1 activator, resveratrol. Molecular docking suggested LEU202, LEU206, ILE223, and ASN226 as amino acid residues played roles in the binding. Additionally, the carbonyl group of both acetate compounds (i.e., geranyl acetate and linalyl acetate) was noted to be essential for hydrogen bond formation with a key amino acid residue ASN226. The in silico ADMET predictions also revealed that these compounds are drug-likeness possessing required properties to be further developed as oral CNS drugs with desirable safety (without hepatotoxicity, immunotoxicity, and carcinogenicity). Accordingly, these coriander-derived compounds might be promising leads to be further developed for the treatment and prevention of NDs. However, further studies regarding the mechanism of action in *in vivo* and clinical trials are still required.

## Electronic supplementary material

Below is the link to the electronic supplementary material.

Supplementary Material 1

## Data Availability

The data and materials that support the findings of this study are available from the corresponding author upon reasonable request.
