# Supplementary material for: Neuroprotective Properties of Coriander-Derived Compounds on Neuronal Cell Damage under Oxidative Stress-Induced SH-SY5Y Neuroblastoma and in Silico ADMET Analysis
Source: Neurochem Res. 2024 Sep 19;49(12):3308–25. doi: 10.1007/s11064-024-04239-0 (PMC11502562; doi:10.1007/s11064-024-04239-0)
Supplement: Supplementary file 1 — Supplementary Material 1 [file 11064_2024_4239_MOESM1_ESM.docx]

Supplementary Material

Neuroprotective Properties of Coriander-Derived Compounds on Neuronal Cell Damage under Oxidative Stress-Induced SH-SY5Y Neuroblastoma and *in Silico* ADMET Analysis

Papitcha Jongwachirachai^1^, Waralee Ruankham^1^, Setthawut Apiraksattayakul^1^, Saruta Intharakham^1^, Veda Prachayasittikul^1^, Wilasinee Suwanjang^1^, Virapong Prachayasittikul^2^, Supaluk Prachayasittikul^1^ and Kamonrat Phopin^1,2*^

^1^Center for Research Innovation and Biomedical Informatics, Faculty of Medical Technology, Mahidol University, Bangkok 10700, Thailand

^2^Department of Clinical Microbiology and Applied Technology, Faculty of Medical Technology, Mahidol University, Bangkok 10700, Thailand

*** Correspondence:**Kamonrat Phopin
kamonrat.php@mahidol.edu , kamonrat.php@mahidol.ac.th

**
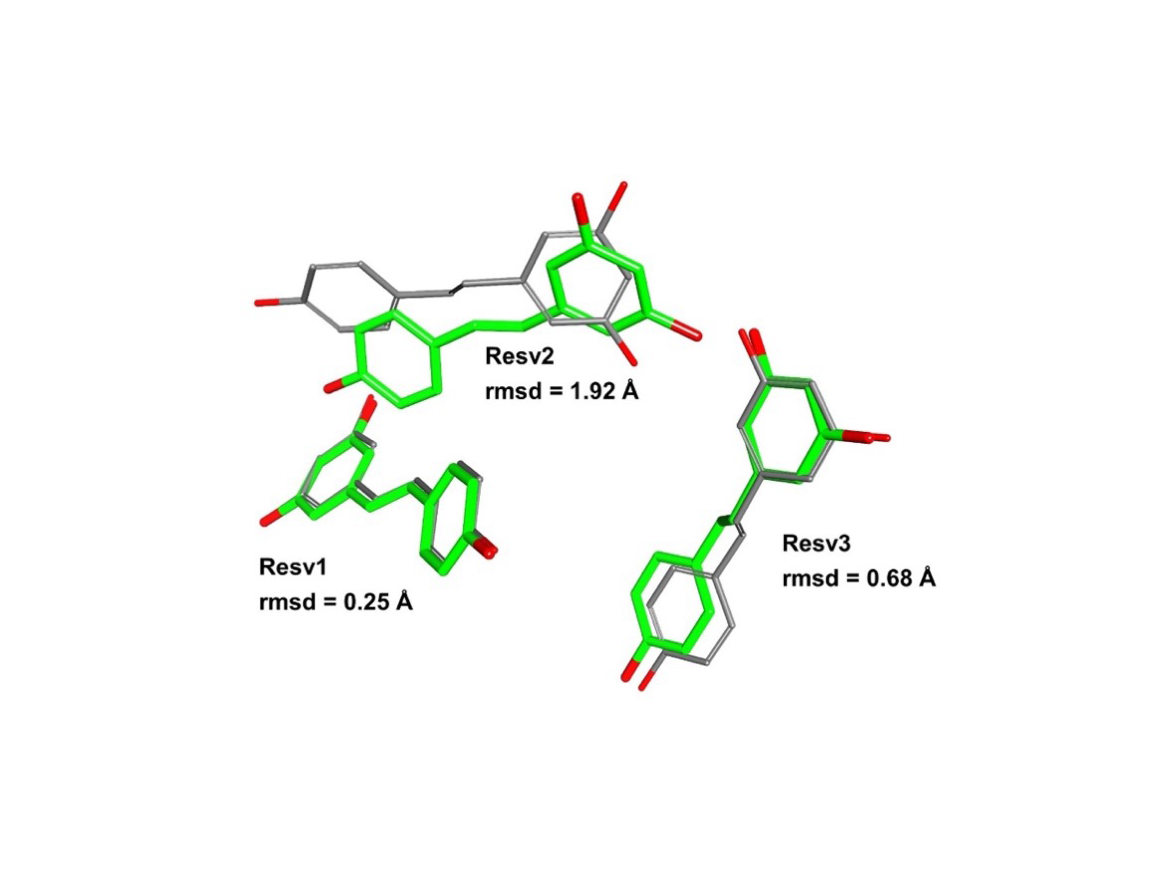
**

**Figure S1** Superimposition of our redocked poses of three resveratrol structures (carbon in green) with their corresponding structure (carbon in grey) observed in the crystal structure (PDB i.d. 5BTR). Root mean square deviations (rmsd) between redocked and crystallographic structures were indicated.
